# Generation of Engineered Human Myocardium in a Multi-well Format

**DOI:** 10.1016/j.xpro.2020.100032

**Published:** 2020-06-03

**Authors:** Malte Tiburcy, Tim Meyer, Norman Y. Liaw, Wolfram-Hubertus Zimmermann

**Affiliations:** 1 Institute of Pharmacology and Toxicology, University Medical Center Göttingen, Göttingen, Germany; 2 DZHK (German Center for Cardiovascular Research), Partner Site Göttingen, Göttingen, Germany; 3 Cluster of Excellence "Multiscale Bioimaging: from Molecular Machines to Networks of Excitable Cells" (MBExC), University of Göttingen, Göttingen, Germany

## Abstract

This protocol describes a robust method for the generation of engineered human myocardium (EHM) from pluripotent stem cells (PSCs) in a multi-well plate under defined, serum-free conditions. By parallel culture of up to 48 EHM in one plate, contractile heart muscle can be obtained to serve numerous applications, including drug screening and disease modelling. This protocol has been successfully applied to human embryonic stem (HES) cell- and induced PSC-derived cardiomyocytes, subtype-specific, i.e., atrial and ventricular, and commercially available cardiomyocyte preparations. For complete details on the use and execution of this protocol, please refer to Tiburcy et al. (2017).

## Highlights

• A robust collagen-based protocol to generate engineered human myocardium (EHM)
• EHM derived from pluripotent stem cell (PSC)-derived cardiomyocytes and fibroblasts
• Enables culture of up to 48 EHM in one plate to enable large-scale screening applications
• Usable with cardiomyocytes and fibroblasts from several sources and of different subtypes

## Graphical Abstract

**Figure:**
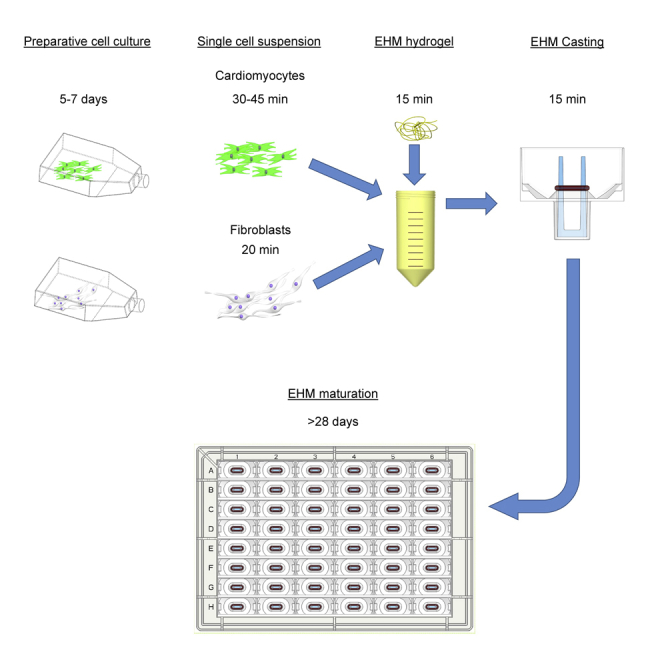

## BEFORE YOU BEGIN

The generation of engineered human myocardium (EHM) requires human PSC-derived cardiomyocytes and fibroblasts and follows these major steps:

1. Cardiomyocyte preparation: We typically use cardiomyocytes from human PSCs 18 to 25 days after the start of directed differentiation. Cardiomyocytes should have a high purity (>90%). Cardiomyocytes from different monolayer and embryoid body protocols have been successfully used for EHM generation (Borchert et al., 2017, Chen et al., 2015, Cyganek et al., 2018, Kattman et al., 2011, Riegler et al., 2015, Tiburcy et al., 2017). In addition, cryo-preserved cardiomyocytes, for example from commercial vendors, may be applied. For optimal standardization of the EHM protocol we recommend to re-plate either fresh or frozen cardiomyocytes as monolayer culture prior to their defined application (steps 1–4). If cardiomyocytes are derived in monolayer differentiation you may directly proceed to step 5.
2. Fibroblast preparation (steps 14–18): We typically use human foreskin fibroblasts (HFF) for practical reasons (availability from for example ATCC as readily expandable cultures, see Key Resources Table) and standardization.

***Alternatives:*** Primary cardiac fibroblasts (see Key Resources Table) or PSC-derived fibroblasts will similarly work and may be advantageous under certain experimental conditions, e.g., disease modeling.

3. Cell mix and hydrogel preparation (steps 28–35): For optimal results, EHM are constructed from 0.5 million cell mixtures (consisting of 70% cardiomyocytes and 30% fibroblasts) in a final reconstitution volume of 180 μl. The use of commercially available 48-well plates permits casting and culture in a one-step procedure with a defined circular geometry and exposure to mechanical loading for video-optic monitoring of EHM formation and function.

***Note:*** All procedures are performed in Class II biosafety hoods located within laboratories rated at biosafety level 1 or 2 (depending on the virus status of PSC lines and local regulations). Approval for work with PSCs and their derivatives must be obtained and performed in accordance with respective legal and ethical regulations. All cultures are maintained in a humidified incubator at 37°C and 5% CO_2_.

## KEY RESOURCES TABLE

**Table:** 

REAGENT or RESOURCE	SOURCE	IDENTIFIER
**Chemicals, Peptides, and Recombinant Proteins**		

Albumin, recombinant human	Sigma-Aldrich	Cat. #A9731
B-27 Supplement (50x)	Thermo Fisher Scientific	Cat. #17504044
B-27 Supplement minus insulin (50x)	Thermo Fisher Scientific	Cat. #A18956-01
Collagen (Acid Solubilized Telo Collagen)	Collagen Solutions	Cat. #FS22024
FGF-2	Peprotech	Cat. #AF-100-18B
IGF-1	Peprotech	Cat. #AF-100-11
L-ascorbic acid 2-phosphate	Sigma-Aldrich	Cat. #A8960
Matrigel (Growth Factor Reduced)	BD	Cat. #354230
MEM non-essential amino acid solution	Thermo Fisher Scientific	Cat. #11140035
RPMI powder	Thermo Fisher Scientific	Cat. #51800-035
Sodium pyruvate	Thermo Fisher Scientific	Cat. #11360070
StemPro Accutase Cell Dissociation Reagent	Thermo Fisher Scientific	Cat. #A1110501
TGF-β1	Peprotech	Cat. #AF-100-21C
TrypLE Express	Thermo Fisher Scientific	Cat. #12605010
VEGF_165_	Peprotech	Cat. #AF-100-20
Versene solution (0.48 mmol/L EDTA)	Thermo Fisher Scientific	Cat. #15040033
Y27632 (ROCK inhibitor)	Stemgent	Cat. #04-0012-10

**Experimental Models: Cell Lines**		

HFF-1	ATCC	Cat. #SCRC-1041
NHCF-V	Lonza	Cat. #CC-2904
HCF	Promocell	Cat. #C-12375

**Other**		

48 EHM multi-well plate	myriamed GmbH	myrPlate

## MATERIALS AND EQUIPMENT

For EHM generation we recommend using 48 EHM multi-well plates which allow parallel EHM casting, culture, and analysis in one platform.

***Alternatives:*** We have reported other methods to generate EHM in detail before (Soong et al., 2012, Tiburcy et al., 2014). The compatibility of this EHM protocol with other tissue culture platforms has to be determined by the user.

**Table:** EHM Medium (EHMM)

Reagent	Final Concentration	Volume (mL)
Iscove’s with Glutamax	n/a	500
MEM non-essential amino acid solution	n/a	5.2
B-27 supplement minus insulin	n/a	20
L-ascorbic acid 2-phosphate (300 mM)	300 μM	0.52
IGF-1 (100 μg/mL)	100 ng/mL	0.52
VEGF_165_ (5 μg/mL)	5 ng/mL	0.52
FGF-2 (10 μg/mL)	10 ng/mL	0.52
**Total**	**n/a**	**527.8**

***Optional:*** Add antibiotics such as 100 U/mL penicillin and 100 μg/mL streptomycin. Store at 4°C for up to 14 days.

**Table:** EHM Medium with TGF-β1 (EHMM+T)

Reagent	Final Concentration	Volume (mL)
EHMM	n/a	49.95
TGF-β1 (5 µg/mL)	5 ng/mL	0.05
**Total**	**n/a**	**50**

***Note:*** TGF-β1 is added to support EHM condensation typically for the first 3 culture days. Store at 4°C for up to 14 days.

**Table:** Cardio Culture Medium (CCM)

Reagent	Final Concentration	Volume (mL)
RPMI 1640 with Glutamax	n/a	500
Sodium pyruvate	1 mM	5.1
B-27 supplement	n/a	10
L-ascorbic acid 2-phosphate (300 mM)	200 μM	0.35
**Total**	**n/a**	**515.45**

***Optional:*** Add antibiotics such as 100 U/mL penicillin and 100 μg/mL streptomycin. Store at 4°C for up to 14 days.

**Table:** Cardio Recovery Medium (CRM)

Reagent	Final Concentration	Volume (mL)
Cardio medium	n/a	49.95
Y27632 (10 mM), added freshly before use	10 μM	0.05
**Total**	**n/a**	**50**

***Optional:*** Add antibiotics such as 100 U/mL penicillin and 100 μg/mL streptomycin.

### RPMI (10x)

Dissolve 104 mg/mL of RPMI powder in ddH_2_O under constant rotation at 37°C for 1 h. Sterilize by filtration. Store at -20°C for up to 12 months.

**Table:** RPMI (2x)

Reagent	Final Concentration	Volume (mL)
RPMI 10x	n/a	2
B-27 Supplement minus insulin	n/a	0.8
ddH_2_O	n/a	7.2
**Total**	**n/a**	**10**

***Optional:*** Add antibiotics such as 100 U/mL penicillin and 100 μg/mL streptomycin. Store at 4°C for up to 14 days.

### IGF-1 (100 μg/mL)

Dissolve IGF-1 in PBS containing 0.1% recombinant albumin to obtain a 100 μg/mL stock solution. Aliquot and store at -20°C for up to 12 months. Once thawed, keep at 4°C for up to one week.

### VEGF_165_ (5 μg/mL)

Dissolve VEGF_165_ in PBS containing 0.1% recombinant albumin to obtain a 5 μg/mL stock solution. Aliquot and store at -20°C for up to 12 months. Once thawed, keep at 4°C for up to one week.

### FGF-2 (10 μg/mL)

Dissolve FGF-2 in PBS containing 0.1% recombinant albumin to obtain a 10 μg/mL stock solution. Aliquot and store at -20°C for up to 12 months. Once thawed, keep at 4°C for up to one week.

### TGF-β1 (5 μg/mL)

Dissolve TGF-β1 in PBS containing 0.1% recombinant albumin to obtain a 5 μg/mL stock solution. Aliquot and store at -20°C for up to 12 months. Once thawed, keep at 4°C for up to one week.

### L-Ascorbic Acid 2-Phosphate (300 mM)

Dissolve 4.3 g of L-ascorbic acid 2-phosphate in 50 mL ddH_2_O to obtain a 300 mmol/L stock solution. Mix until dissolved and sterilize by filtration. Aliquot and store at -20°C for up to 12 months.

### NaOH (0.1 N)

Dilute 1 N NaOH 1:9 in ddH_2_O. Sterilize by filtration.

## STEP-BY-STEP METHOD DETAILS

### Preparative Cardiomyocyte Monolayer Culture

**Timing: 5–7 days**

We recommend this step for optimal standardization of cardiomyocyte cultures before EHM generation: in particular, if cryo-preserved cardiomyocytes are used or cardiomyocytes that have been enzymatically dispersed (steps 5–13 or alternative digestion protocols). Cardiomyocytes should be cultured in monolayer for 5–7 days before proceeding to EHM generation. If cardiomyocytes are differentiated in monolayer you may directly proceed to step 5.

***Note:*** Volumes are given for a T75 cell culture flask. For different culture formats (e.g., 6-well plates), volumes need to be adjusted according to surface area.

1. Aliquot Matrigel for coating
a. Thaw Matrigel (7–10 mg/mL) for 16–24 hrs on ice at 4°C.
b. Mix Matrigel well by several slow inversions of the bottle, leave on ice.
c. Distribute Matrigel in 250 μL aliquots into 50 mL centrifuge tubes on ice using ice-cold pipette tips.
d. Freeze aliquots at -20°C and store for up to 12 months.
2. Matrigel coating
a. Resuspend a frozen Matrigel aliquot in 29.75 mL ice cold PBS to obtain the working dilution of 1:120.
b. Add 6 mL to a T75 flask and ensure uniform distribution.
c. Coat flask for a minimum of 60 min at 20–25°C or store at 4°C for up to 14 days.

**Pause Point:** Matrigel-coated flasks can be stored for 14 days at 4°C. Drying of the coated surface should be prevented.

3. Cardiomyocyte seeding after enzymatic dispersion
a. Warm Matrigel-coated flask to 20–25°C, warm Cardio Culture Medium to 37°C.
b. Centrifuge cardiomyocytes in Cardio Recovery Medium (200 x g for 7 min at 4°C).
c. Aspirate Matrigel from pre-coated flask and add 5 mL of Cardio Culture Medium to flask
d. Carefully aspirate the supernatant and flick the tube to dislodge the pellet. Resuspend the cells in 10 mL of Cardio Culture Medium
e. Seed into the flask with a density of 0.1–0.2x10E6/cm^2^.
f. Replace 15 mL Cardio Culture Medium every other day.
4. Thawing of cryo-preserved cardiomyocytes
a. Warm Cardio Recovery Medium and a Matrigel-coated flask to 20–25°C, warm Cardio Culture Medium to 37°C.
b. Remove cryo-vials containing cardiomyocytes from liquid nitrogen and thaw at 37°C for 3 min.
c. Desinfect cryo-vials with 70% EtOH
d. Remove cell suspension from cryo-vial with a 2 mL serological pipette and transfer to a centrifuge tube. Wash cryo-vial with 1 mL of Cardio Recovery Medium and add dropwise to the cells.
e. Slowly add another 8 mL of Cardio Recovery Medium.
f. Centrifuge the tube at 200 x g for 7 min at 4°C.

**CRITICAL:** At this stage, the cells are very sensitive; handle them carefully and avoid extensive pipetting. Use a serological pipette with a wide tip to minimize mechanical disruption of cells.

g. Carefully aspirate the supernatant and flick the tube to dislodge the pellet. Resuspend the cells in 10 mL of Cardio Culture Medium.
h. Strain the suspension through a 70 μm mesh cell strainer.
i. Count the cells using an automated cell counter or hemocytometer.
j. Aspirate Matrigel from pre-coated flask and add 5 mL of Cardio Culture Medium to flask
k. Seed the cardiomyocyte suspension into the flask with a density of 0.1–0.2x10E6/cm^2^.
l. Replace 15 mL Cardio Culture Medium every other day.

### Enzymatic Dispersion of Human PSC-Derived Cardiomyocytes

**Timing: 30–45 min**

This step establishes a single cell suspension of PSC-derived cardiomyocytes for EHM generation.

**CRITICAL:** This protocol has been optimized for cardiomyocyte monolayer cultures. We typically use cardiomyocytes between days 18–25 of differentiation. For other cardiomyocyte differentiation protocols, e.g. 3D embryoid bodies, alternative digestion protocols have been published (Chen et al., 2015, Kattman et al., 2011, Tiburcy et al., 2017). For optimal standardization we recommend to plate cardiomyocytes in monolayer culture first (steps 1–3) and then use this digestion protocol when making EHM.

***Note:*** Volumes are given for a T75 cell culture flask. For different culture formats (e.g., 6-well plates), volumes need to be adjusted according to culture plate surface area.

**Figure 1:**
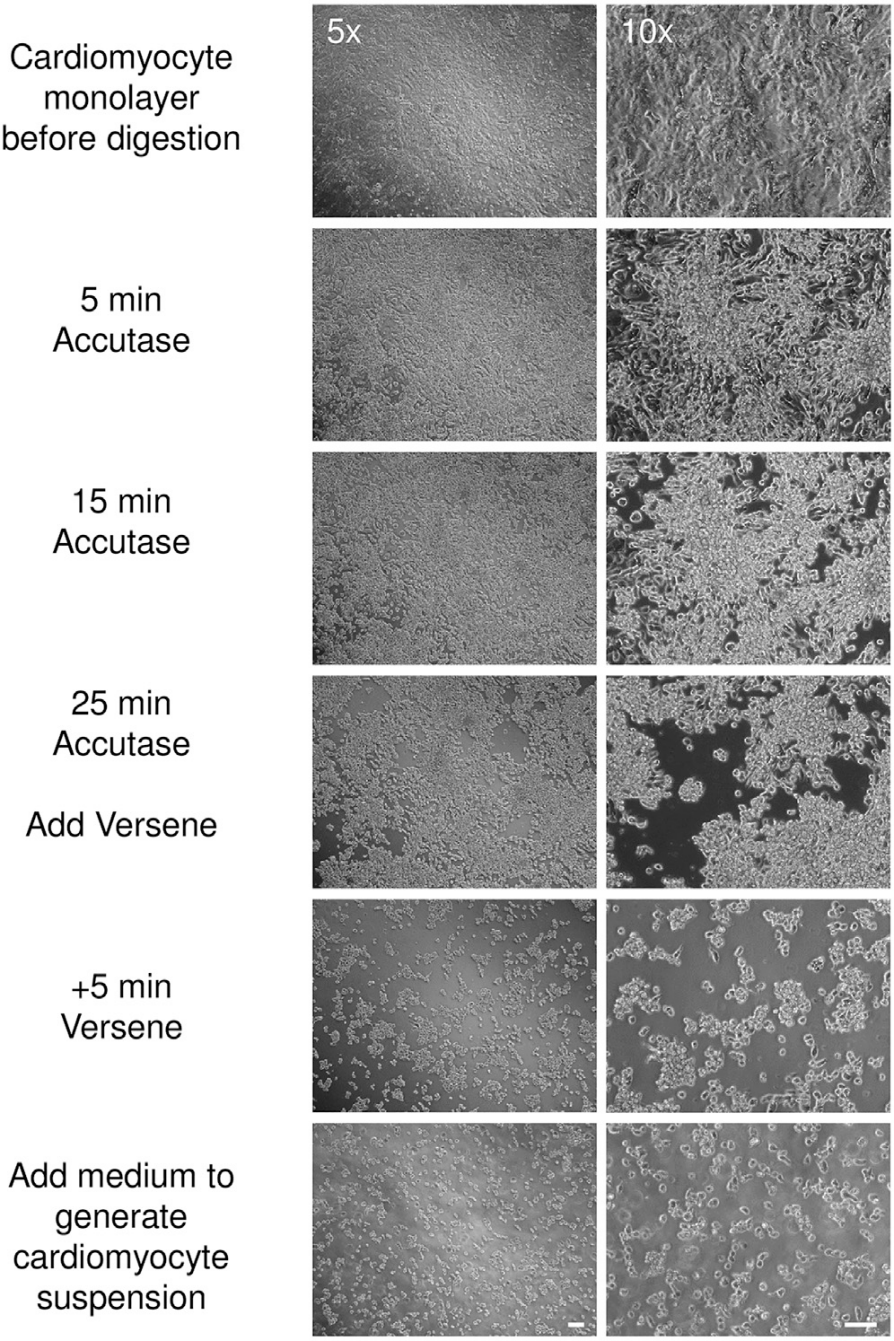
Enzymatic Dispersion of Monolayer Cardiomyocytes Brightfield images of monolayer cardiomyocytes (cultured according to steps 19–23) at indicated stages of enzymatic dispersion. Note, that Versene should be added once larger aggregates of cardiomyocytes lift off the culture plate surface. Bar graph: 50 μm.

***Note:*** Depending on cell density, culture conditions, and time point of differentiation, this may occur after 15 min, but may take up to 45 min. Check detachment repeatedly under the microscope (Figure 1).

***Note:*** During this incubation time, preparation of fibroblasts (see steps 22–26) can be commenced.

5. Warm Cardio Recovery Medium, PBS, and Accutase to 20–25°C. Warm Versene to 37°C.
6. Aspirate medium from flask with human PSC-derived cardiomyocytes.
7. Add 6 mL of Accutase to the cells and incubate for 15–45 min at 20–25°C until cells start to visibly detach (Figure 1).

8. Add 6 mL of Versene to the Accutase and incubate at 20–25°C for a further 5 min.
9. Stop the digestion by adding 12 mL of Cardio Recovery Medium to the Accutase/Versene mix. Pipette up and down gently with a 10 mL serological pipette to ensure a single cell suspension, and transfer cells to a fresh collection tube.
10. Wash flask once more with 6 mL of Cardio Recovery Medium and add to the collection tube. Centrifuge the cells at 200 x g for 7 min at 4°C.

**CRITICAL**: At this stage, the cells are very sensitive; handle them carefully and avoid extensive pipetting. Use a serological pipette with a wide tip to minimize mechanical disruption of cells.

11. Carefully aspirate the supernatant and flick the tube to dislodge the pellet. Resuspend the cells in 10 mL of Cardio Recovery Medium.
12. Strain the suspension through a 70 μm mesh cell strainer.
13. Count the cells using an automated cell counter or hemocytometer. Leave the cells on ice.

### Enzymatic Dispersion of Human Fibroblasts

**Timing: 20 min**

The culture of human foreskin fibroblasts has been described in detail (Schlick et al., 2019). This step establishes a single cell suspension of fibroblasts for EHM generation.

***Alternatives:*** Fibroblasts from other sources including PSC-derived fibroblasts can be used.

***Note:*** Volumes are given for a T75 cell culture flask. For different culture formats (e.g., 6-well plates), volumes need to be adjusted by scaling to surface area.

14. Warm Cardio Culture Medium and TrypLE Express to 37°C.
15. Wash cells with 6 mL TrypLE Express, aspirate and add 6 mL of TrypLE Express again. Incubate at 37°C for 5–10 min.
16. Stop the digestion by adding 12 mL of Cardio Culture Medium.
17. Pipette up and down, and pass the cell suspension through a 70 μm mesh cell strainer into a fresh 50 mL centrifuge tube to remove excess extracellular matrix.
18. Count the cells using an automated cell counter or hemocytometer. Leave the cells on ice.

### Cryo-Preserved Human Fibroblasts (Alternative)

**Timing: 15 min**

EHM can be made directly from cryo-preserved fibroblasts. The advantage of this approach is that fibroblast properties are very standardized over experimental series if the same pool of fibroblasts is used. The following steps describe the preparation of cryo-preserved fibroblasts for EHM generation.

1. Warm Cardio Culture Medium to 15–25°C.
2. Remove cryo-vials containing fibroblasts from liquid nitrogen and thaw at 37°C for 3 min.
3. Disinfect cryo-vial with 70% EtOH.
4. Remove cell suspension from cryo-vial with a 2 mL serological pipette and transfer to a tube. Wash cryo-vial with 1 mL of Cardio Culture Medium, add dropwise to the cells.
5. Slowly add another 8 mL of Cardio Culture Medium.
6. Centrifuge the tube at 300 x g for 5 min at 4°C.
7. Carefully aspirate the supernatant and resuspend the cells in 10 mL of Cardio Culture Medium.
8. Strain the suspension through a 70 μm mesh cell strainer.
9. Count the cells using an automated cell counter or hemocytometer. Leave the cells on ice.

### Generation of EHM

**Timing: 1.5 h**

In this step cardiomyocytes (from step 13) and fibroblasts (from step 18 or 27) are combined into a cell mix and then reconstituted in a collagen hydrogel. The cell-hydrogel mixture is cast into molds and left to solidify (condense) for 1 hr in an incubator. This step is completed by adding medium to the condensed EHM hydrogels.

19. Determine the number of EHM to be prepared based on the number of cardiomyocytes and fibroblasts prepared on ice (live cells):
20. Mix the appropriate number of cardiomyocytes and fibroblasts into a new tube according to Table 1 and centrifuge tube at 200 x g for 7 min at 4°C.

**Table 1:** Preparation of Cell Mix (Including a 10% Surplus to Account for Pipetting Error)

EHM Number:	1	6	48
Cardiomyocytes	0.385 million	2.31 million	18.48 million
Fibroblasts	0.165 million	0.99 million	7.92 million
21. Aspirate the supernatant and resuspend the cell mix in 4°C cold EHMM (volume according to cell mix in Table 2). Leave the cell mix on ice.

***Note:*** A collagen concentration of 0.3 mg/EHM is recommended. A collagen stock solution containing 6-7 mg/mL is recommended. Volumes of the other EHM components have to be adapted according to the volume of the collagen stock solution needed to obtain on optimal EHM collagen content. The NaOH amount may have to be adapted individually for optimal pH neutralization (see Critical point below).

22. Prepare the EHM hydrogel on ice in the exact order listed in Table 2. To a 50 mL centrifuge tube on ice:
a. Add the acid soluble collagen.
b. Add the 2x RPMI and mix by swirling the tube.
c. Add 0.1 N NaOH and mix by swirling the tube.
d. Add the cell mix.

**Table 2:** Preparation of EHM Hydrogel (Including a 10% Surplus Accounting for Pipetting Errors)

	Volume (μl)		
EHM number:	1	6	48
Collagen (6.84 mg/mL)	44	264	2,112
2x RPMI	44	264	2,112
0.1 N NaOH	6	36	288
Cell mix in EHMM	104	624	4,992
**Total volume**	**198**	**1,188**	**9,504**

Mix the entire suspension well by triturating >10 times with a serological pipette.

**CRITICAL:** pH neutralization of the acid soluble collagen must be confirmed by inspection of the phenol red indicator transition from yellow to pink. At this point, the condensation (solidification) of collagen will commence and must not be disrupted so the following steps should be performed swiftly.

***Note:*** As collagen is very viscous, pipette slowly to avoid air bubble formation.

**Figure 2:**
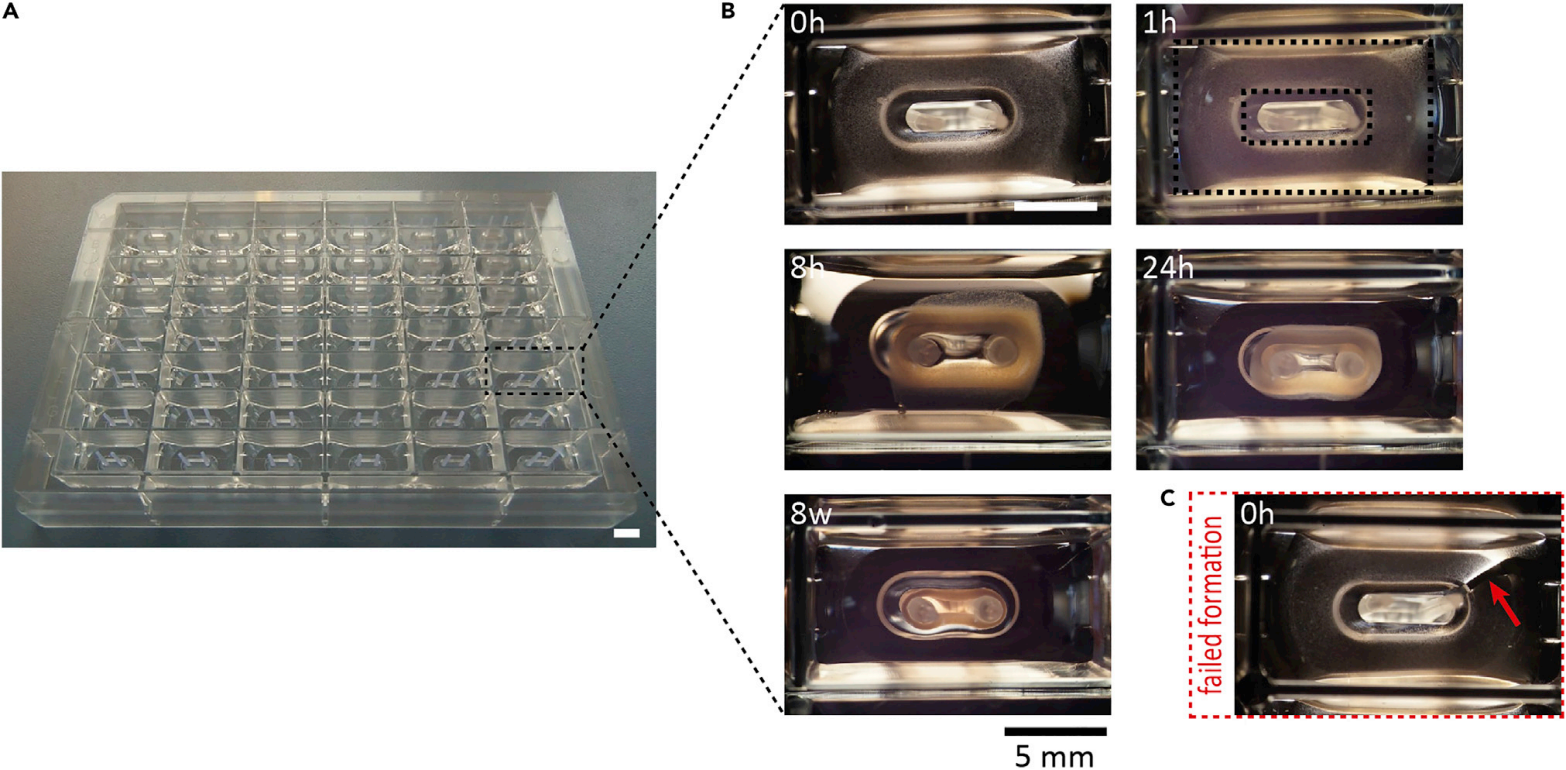
Generation and Parallel Culture of 48 EHM in a Multi-well Format (A) 48-well EHM plate used for the construction of EHM. (B) EHM forming between two poles over time. The prepared hydrogel becomes opaque after 1 h (dashed boxes indicate inner and outer boundary of the hydrogel) and compacts notably by 24 h. After four weeks in culture, EHM are typically analyzed. (C) Breaks in the hydrogel (arrow) will prevent correct EHM formation. Bar graph: 5 mm.

***Note:*** If breaks in the hydrogel occur (Figure 2C arrow), an intact EHM loop will not form.

23. Distribute 180 μl of EHM hydrogel with a 1 mL pipette tip evenly into each well of a 48-well EHM plate (Figure 2A). Ensure an even distribution of hydrogel in each well by lightly tapping the plate (Figure 2B).

24. Carefully transfer the 48-well plate to the incubator. Let the reconstitution mix settle for 1 h, after which it will appear opaque (Figure 2B).
25. Carefully add 600 μl of 37°C EHMM+T per well and incubate for 24 h.
26. Exchange 500 μl of EHMM+T per well daily for the first three days of EHM formation. Afterwards, feed with EHMM (without TGF-β1) until analysis.

***Note:*** After the initial phase of cell independent hydrogel condensation, fibroblasts will start to compact the cell-hydrogel mixture. Within 24 h, EHM should have almost completely formed a loop structure (Figure 2B). Spontaneous contractions of the EHM loops can typically be observed after 3 days of EHM formation and will noticeably increase over time in culture.

### Monitoring of Contractile Function in Culture

**Timing: 5 min**

**Figure 3:**
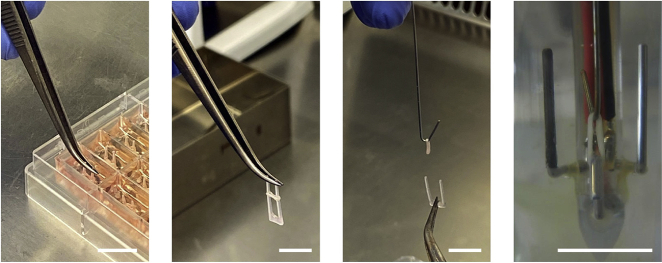
Removal of EHM from Multi-well Plate for Individual Culture and Analyses EHM can be easily removed from multi-well plate with sterile forceps for continuous individual culture or analysis. In this example the transfer to a metal hook for a contractile force measurement under isometric conditions and electrical field stimulation (note electrodes on left and right of the isometrically suspended EHM) is shown. Bar graph: 1 cm.

EHM culture is typically performed for 28 days, but can be further extended. We have tested EHM cultures in a multi-well format for up to 12 months without an apparent loss of function. Functional assessment of EHM can be performed by video-optic analyses or in a standard organ bath set-up under isometric conditions after removal of EHM from the multi-well plate (Figure 3) at any day during culture.

27. Exchange 500 μl of EHMM per well (without TGF-β1) daily.

***Note:*** An “every-other-day” feeding interval may be applied once or twice a week.

## EXPECTED OUTCOMES

After successful formation of EHM first contractions are typically observed after 3 days. Force generation, as detected by rhythmic pole deflection, will increase notably within the first 2 weeks to reach robust levels by week 4. Nevertheless, the magnitude of pole deflection may further increase and cultures can be maintained for up to 1 year (longest time tested). Spontaneous contraction rate typically decreases with time in culture and can cease completely, requiring electrical stimulation for contractile studies. Apart from the functional analyses EHM can be easily removed from the plate by pulling the flexible holders out to continue with downstream analyses (Figure 3). For details on the use and in-depth characterization of the resulting EHM, please consult Tiburcy et al., 2017 and Cyganek et al., 2018.

## LIMITATIONS

Contractility measurements in the multi-well plate format require spontaneously beating EHM and are not performed under defined isometric conditions. Spontaneous beating rate decreases with time in culture. Thus, external electrical stimulation may be required for functional analyses in the multi-well format. Alternatively, remove EHM to perform assessments of contractile function under isometric conditions and electrical stimulation individually in standard organ bath set-ups (e.g., Radnoti 8 Unit Tissue Organ Bath System).

## TROUBLESHOOTING

### Problem

EHM disrupted after initial culture medium addition, i.e., 1 h after casting.

### Potential Solution

At this stage care must be taken to not physically disrupt the hydrogel. Do not pipette culture medium directly onto the condensing EHM. Add culture medium slowly via the well wall.

### Problem

EHM do not condense within 1 h after casting.

### Potential Solution

Hydrogel condensation is a material intrinsic, i.e., largely cell independent, property of the collagen type I hydrogel (refer for details to Schlick et al. 2019). Care must be taken to not disrupt condensation by quickly casting the pH neutralized reconstitution mixture. In addition, the collagen hydrogel should be tested as to its condensation properties before use in EHM generation. Testing different suppliers and batches may be required for optimal results. Acetic acid solubilized collagen with a collagen concentration of >4 mg/mL gives best results. Care must be taken to store and handling of collagen at 4±2°C; collagen must never be frozen or handled at room temperature.

### Problem

EHM do not compact within 3 days after casting.

### Potential Solution

The compaction process is a function of the fibroblast component. Ensure fibroblasts viability and sufficient quantity preferably 30% of input cells. Optimal fibroblast content may have to be adapted depending on the source of fibroblasts. A too high fibroblast content will result in massive EHM compaction with a steep increase in EHM stiffness (Young’s Modulus) and inhibit EHM contractility; a too low fibroblast content will result in a lack of EHM compaction with negligible or no contractile force development (refer to Tiburcy et al., 2017 for details).

### Problem

EHM do not contract visibly within 2 weeks after casting.

### Potential Solution

EHM contractility is a function of the cardiomyocyte component. Ensure cardiomyocyte viability after enzymatic dispersion and purity at preferably 70% of input cells. Optimal cardiomyocyte content may have to be adapted depending on the source of cardiomyocytes. Too low or too high cardiomyocyte purity will result in suboptimal or no contractile performance (refer to Tiburcy et al., 2017 for details).

### Problem

EHM do not contract uniformly in the 48-well plate.

### Potential Solution

Casting procedure may have to be trained to ensure completion of the casting from the prepared reconstitution mixture within ideally 5 min. Note that the reconstitution mixture will start to condense after pH neutralization and that this process must not be inappropriately disrupted. However, ensure proper mixing of cells in the prepared reconstitution mixture by trituration (10x on ice) before casting. EHM may stop to contract spontaneously after extended culture, but will remain contractile upon external electrical stimulation.

### Problem

EHM do not form a homogeneous loop.

### Potential Solution

Ensure continuous pipetting of a well-mixed EHM reconstitution mixture into the mold. Breaks or inhomogenous distribution of EHM reconstitution mixture must be avoided.
